# Polymer 3D Printing Review: Materials, Process, and Design Strategies for Medical Applications

**DOI:** 10.3390/polym13091499

**Published:** 2021-05-06

**Authors:** Amit M. E. Arefin, Nava Raj Khatri, Nitin Kulkarni, Paul F. Egan

**Affiliations:** Mechanical Engineering, Texas Tech University, Lubbock, TX 79409, USA; amit.arefin@ttu.edu (A.M.E.A.); nakhatri@ttu.edu (N.R.K.); nitin.kulkarni@ttu.edu (N.K.)

**Keywords:** 3D printing, additive manufacturing, materials, polymers, design, lattices, mechanics, simulation, engineering, medical

## Abstract

Polymer 3D printing is an emerging technology with recent research translating towards increased use in industry, particularly in medical fields. Polymer printing is advantageous because it enables printing low-cost functional parts with diverse properties and capabilities. Here, we provide a review of recent research advances for polymer 3D printing by investigating research related to materials, processes, and design strategies for medical applications. Research in materials has led to the development of polymers with advantageous characteristics for mechanics and biocompatibility, with tuning of mechanical properties achieved by altering printing process parameters. Suitable polymer printing processes include extrusion, resin, and powder 3D printing, which enable directed material deposition for the design of advantageous and customized architectures. Design strategies, such as hierarchical distribution of materials, enable balancing of conflicting properties, such as mechanical and biological needs for tissue scaffolds. Further medical applications reviewed include safety equipment, dental implants, and drug delivery systems, with findings suggesting a need for improved design methods to navigate the complex decision space enabled by 3D printing. Further research across these areas will lead to continued improvement of 3D-printed design performance that is essential for advancing frontiers across engineering and medicine.

## 1. Introduction

**Figure 1:**
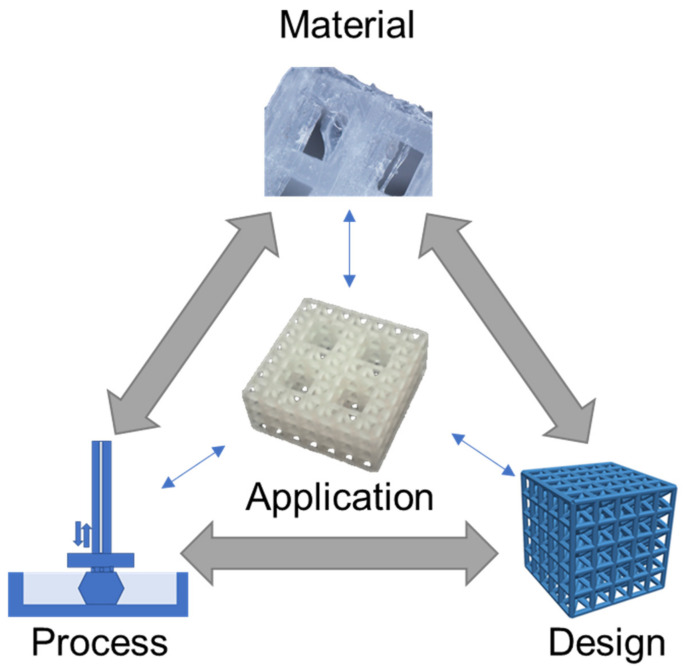
Material, process, and design considerations for medical applications, illustrated for a tissue scaffold example [17]. Images adapted with permission.

Polymer 3D (three-dimensional) printing has advanced rapidly in recent years with many areas of research now translating to engineered products, especially in medical fields [1,2,3,4]. Polymer printing is advantageous for a broad range of medical areas that benefit from the diversity of polymer material characteristics and processing approaches [5,6,7,8]. 3D printing is a highly desirable fabrication approach because it enables the construction of designs with complex geometries and architectures that are not possible with conventional manufacturing processes. For instance, tissue scaffold structures fabricated with polyjet and stereolithography printing can achieve hierarchical forms that mimic bone, thereby providing a mechanical and biological niche to support tissue regeneration [9,10]. Additionally, the selection of polymers has advantages over metal printing approaches, that result in metal implants that do not degrade in the body and lead to mechanical issues such as stress shielding [11]. In areas of safety equipment, polymer-printed lattices achieve efficient energy absorption with a rapid fabrication process that bypasses the supply chain limitations of bulk manufacturing [12,13]. Polymer printing is possible using extrusion, resin, and powder 3D printing processes that provide versatility for material selection and supporting designs with diverse architectures, responses, and layouts [14,15]. Because of the large design space offered by 3D polymer printing, and its opportunities for improving medical applications, we carried out a critical review by considering recent advances in materials, processes, and design strategies that all influence an application’s outcome [16], as illustrated in Figure 1 for a tissue scaffold example.

The Figure 1 schematic highlights a hierarchical tissue scaffold constructed from beam-based unit cells with interconnected considerations in materials, process, and design for ensuring appropriate mechanical and biological functioning [17]. In this example, a design strategy for mimicking the hierarchical structure of bone largely drove the need for a suitable printing process and material selection to support the application. The material choice was dictated by a need for appropriate stiffness to ensure structural integrity while retaining biocompatibility to promote tissue growth, which was fulfilled with a methacrylic acid-based polymer. The printing process requires the formation of layers for building the complicated hierarchical truss structure, which was achieved by stereolithography printing. However, once these factors are selected, there is a need to iterate and refine the structure’s design based on performance variability attributed to uncertainty and part variation in the 3D printing process [9,18].

Comparative studies for tissue scaffolds can achieve widely different design strategies based on different material/printing process decisions. For instance, tissue scaffolds constructed from polycaprolactone (PCL) using fused deposition modeling have more compliant structures with biodegradability, while titanium scaffolds printed with selective laser sintering have a higher stiffness, but no biodegradability [19,20]. These choices then influence the scaffold’s topological design, since it is generally not feasible to print polycaprolactone as a truss-based structure, whereas selective laser sintering processes are able to produce titanium in forms to achieve mechanically efficient truss-based structures that promote high porosity for large void volumes for tissue growth.

Generally, decisions across material, process, and design strategies occur in a nonlinear and integrated fashion that requires careful consideration and knowledge of their relation to an application. Here, materials, process, and design strategies for polymer printing are reviewed in the context of medical applications, with a critical assessment for how each of these decision factors influence applications and one another. Initially, materials are reviewed to highlight their capabilities and properties, with data presented to compare diverse materials available for mechanical applications. Reviews on printing processes include extrusion, resin, and powder printing, which are among the most common approaches for polymer printing, with considerations for how processing influences part fidelity and functionality. The investigation of design strategies provides an overview for organizing processed materials that is advantageous for tuning application performance. Considered applications include prosthetics, safety equipment, and drug delivery, which provide context for how fundamental research in these areas translates to medical scenarios. The review concludes with considerations and challenges for researchers to consider as polymer printing continues advancing, with a particular need for new methodological design approaches to improve engineering outcomes in medicine.

## 2. Material Capabilities

Material capabilities of polymers for 3D printing are informed by their molecular structures, and also depend on a material’s processing during printing. The selection of materials for design applications is often conducted by considering measurable properties, such as mechanical properties, with ranges based on processing and testing methods that provide further complications in predicting part performance during the system design.

### 2.1. Material Structure

There is a broad range of polymer materials for 3D printing, with capabilities informed from their molecular structure, with polymers processed in different manners for each printing process. In extrusion processes, thermoplastics are commonly used for 3D printing where they are melted for extrusion followed by hardening after deposition [21]. For example, acrylonitrile butadiene styrene (ABS) is a common thermoplastic that exhibits favorable impact strength and improved chemical resistance compared to pure polystyrene [22]. The properties of ABS are tunable based on the ratio of its three monomers, for instance, its density may range from 1.05 mg/m^3^ to 1.07 mg/m^3^ with resulting tensile moduli from 2.5 GPa to 2.7 GPa. Acrylonitrile styrene acrylate (ASA) is an alternative to ABS with improved heat resistance and exceptional ultraviolet stability [23], while polylactic acid (PLA) is another popular thermoplastic with biocompatibility but a lower glass transition temperature.

PLA is also suitable for further types of printing processes, such as resin curing with stereolithography [24], which enables the construction of more complex part architectures than is generally possible with extrusion processes. Although PLA is biocompatible, there is some concern for toxicity in stereolithography printed PLA because of the addition of photopolymers to the resin solution, which is necessary for cross-linking monomers to form polymers in the presence of ultraviolet light. However, when properly printed and post-processed, resin curing processes have been demonstrated as safe for medical applications, depending on the particular combination of chemical components [25]. These considerations for linking the chemical structure of a polymer to its functioning and printing are essential in pairing printing processes with materials to achieve a desired set of properties for a specified application.

### 2.2. Material Properties

**Figure 2:**
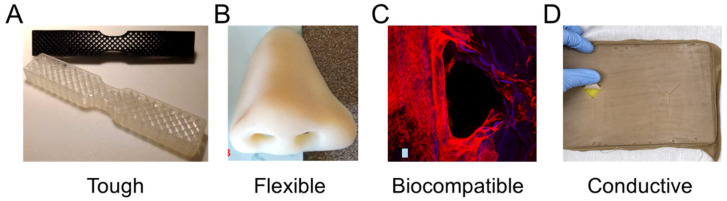
Materials with highlighted properties for (**A**) toughness [26], (**B**) flexibility [28], (**C**) biocompatibility [30], and (**D**) conductivity [31]. Images adapted with permission.

There are diverse material properties necessitated by medical applications that are achievable through 3D printing. Often, medical applications drive the need for specific material capabilities, such as the need for energy absorbing materials in impact resistance, multicolored parts with suitable textures for modeling surgical anatomies, or specified material properties to mimic biological tissues. Figure 2 highlights recent research in medical polymer materials with a focus on mechanical capabilities for toughness [26,27] and flexibility [28,29], biological capabilities for biocompatibility [24,30], and further capabilities such as electrical conductivity [31,32].

Toughness in a material refers to its capability to absorb energy and plastically deform without fracturing, which is calculated from a combination of the material’s strength and ductility. Recently, a 3D-printed tensile bar with crosshatch structures was printed from a tough polyurethane material with comparisons including physically cross-linked Carbothane AC-4095A in pellet form and chemically cross-linked polyurethane with 68A hardness in liquid resin form (Figure 2A) [26]. Results demonstrated elastomeric polyurethanes are relatively tolerant of architectures and notches, which also promotes their use in a variety of design strategies. A further example of toughness for biomedical materials was demonstrated with a methacrylic polymer printed using the resin curing process with a tensile strength of 41 MPa and a general elongation up to 50% before breaking [27]. The material was used for printing a shaft coupling for an assembly without any post-treatment necessary due to the high accuracy of the printing process.

Flexible materials have been constructed recently that are useful as prosthetics, and enable the optimization of a specified form for a person’s unique physiology through scanning and fitting technologies (Figure 2B) [28]. The patient of interest for the study was 27 years old and had a topographic scan of their face that used 3D mapping software to print the nose shape using a Stratasys polyjet printer with TangoPlus flexible material. The TangoPlus material had a 26 to 28 Shore A Hardness, 0.8 to 1.5 MPa tensile strength, and 2 to 4 kg/cm tear resistance, while having a feel similar to rubber. Recoloring was necessary to match the patient’s skin tone. Flexible materials have also been used to print complex structures, such as an Eiffel tower model printed with temperature-stimulated flexible polymer printed using stereolithography [29]. The model distorts at lower temperature and as the temperature increases to 70 °C, the print regains its original form. This temperature-dependent functionality provides possibilities for medical applications with heat-initiated actuation, which could be initiated by body heat or devices.

Biocompatibility is a necessary material property for printed devices that interact with the body, such as hearing aids and retainers, or are implanted in vivo, such as artificial joints or tissue scaffolds. Depending on the application, biocompatibility can have differing criteria, but generally refers to the need for the material to do no harm to the body while facilitating its intended function. For tissue scaffolds, biocompatibility typically refers to a need for non-cytotoxicity, biodegradability, and promotion of tissue growth. Polyjet printing uses Stratasys MED610 material, which is an acrylic-based polymer that has recently had success for printing tissue scaffolds of complex topologies (Figure 2C) [30]. Biological testing was conducted by measuring cell viability using Saos-2 cells that survived, with no difference between the 3D-printed materials and controls after 48 h. Further testing demonstrated growth on tissue scaffold surfaces; however, the growth was limited compared to other tissue engineering materials. An alternative approach is the use of stereolithography for 3D-printed lattices using polylactic acid that can reliably form lattice structures with microscale features [24]. Further testing is required to determine the benefits of 3D-printed polymers to conventional tissue engineering approaches; however, polymers provide immediate advantages over metals due to their ability to degrade safely in vivo.

Electrical conductance is another material property that is useful for medical applications and has been used for fabricated, sensorized tissue analogues through the 3D printing of an organogel. The technology was used to create a suture training pad fabricated with embedded piezoresistive strain sensors and conductive threads as electrodes to quantify the performance of the trainee (Figure 2D) [31]. Fabrication steps included fixing nylon fabric to the bottom of a PLA mold, then pouring and curing skin-colored liquid PDMS, inserting conductive threads into the 3D-printed organogel, encapsulating sensors, adding a fat layer, and cutting the sample to form a suture pad. Further polymer electrical conductivity has been demonstrated with thermoplastics mixed with conductive carbon black fillers for 3D printing a chess rookie that enables turning on an LED light [32]. These printing capabilities enable new types of design applications that could provide feedback in different medical scenarios through embedding sensors in fabricated designs, possibly activating when certain mechanical triggers are reached.

### 2.3. Material Capabilities

Properties of 3D-printed parts are dependent on both their material structure and printing process, and therefore require extensive testing of combinations of materials/process parameters to determine material capabilities for a given application [33]. For instance, a part’s mechanical response when fabricated with fused deposition modeling is alterable based on the printed layer thickness, processing temperature, and orientation [34,35]. In Table 1 a summary is provided that highlights the measured mechanical properties of some common polymer 3D-printed materials tested as solid samples; additional notes in the table provide context for how testing was carried out to provide ranges of process-dependent properties. Material properties include strength- and stiffness-related metrics that are key properties for selecting suitable materials in mechanical applications.

In Table 1, multiple studies are reported for comparisons of ABS materials that all demonstrated similar, but slightly different mechanical properties [34,35,36], such as tensile strength ranging from 15 MPa to 38 MPa. These differences are accounted for in part because of the different processing temperatures and printing parameters used to construct parts, the slightly different proportions of monomers in ABS’s structure, and the tested part’s orientation. For instance, the low tensile strength measurement of 15 MPa for ABS was due to testing in the transverse loading direction compared to the higher measurements of tensile strength closer to 30 MPa based on the build layer orientation. Similar differences were observed for polycarbonate materials based on their processing and chemicals used to manufacture the material [35,36]. One study concluded that a blend of polycarbonate referred to as a bio-based polycarbonate had a slightly higher strength of 65 MPa and significantly higher elastic modulus of 2100 MPa than a polycarbonate manufactured using fossil fuels with 62 MPa strength and 1500 MPa elastic modulus [35]. Polyether ether ketone (PEEK) and polylactic acid (PLA) are commonly used biocompatible materials with relatively high mechanical strength and stiffness among polymers, and are also manufacturable with fused deposition modeling [37,38]. PEEK is generally the more expensive of the two materials with an elastic modulus up to 4100 MPa, while PLA has an elastic modulus of 4400 MPa; both are the highest values among the surveyed Table 1 materials.

Numerous 3D-printed biocompatible materials have been recently investigated for use as bone tissue scaffolds, with several methacrylic/acrylic-based materials included as examples in Table 1 [9,16,17]. These materials were printed with varied resin curing processes and all demonstrated similar elastic moduli around 1500 MPa to 2000 MPa, with some dependency on build orientation. In comparison to the fused deposition modeling parts, these resin prints have a lower stiffness, although their stiffness is tunable based on the curing time per layer and post-processing curing time that has been demonstrated for lattice structures [16]. Overall, the highlighted materials from Table 1 demonstrate how a single material can achieve varied properties based on its processing, and that varied processes enable material selection with similar property ranges. Further considerations for selecting a material/process combination are fabrication accuracies and consistency, which further add complexity to design decisions when selecting a 3D printing approach for a given application.

## 3. Printing Processes

**Figure 3:**
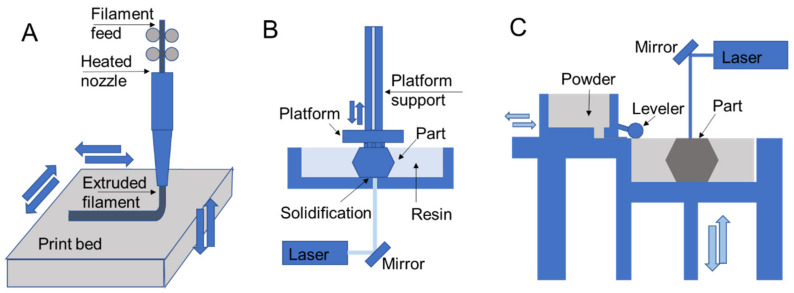
3D printing schematics for (**A**) fused deposition modeling, (**B**) stereolithography, and (**C**) selective laser sintering that are representative of extrusion, resin, and powder processes, respectively.

The most common techniques for polymer 3D printing include extrusion-, resin-, and powder-based processes (Figure 3) [1]. Each type of process enables the additive deposition of layers to form parts and carries out fabrication using unique processing steps that restrict processes to different material selections and capabilities to form designs.

In extrusion processes such as fused deposition modeling, the material is melted and extruded through a nozzle where it is directed for deposition to form part layers (Figure 3A) [41,42]. The filament feed generates nozzle pressure that is used to control material flow during part construction. In direct ink writing, which is another extrusion process, material is pushed through a nozzle according to an applied external shear stress such as air pressure or piston movements [43]. Resin 3D printing relies on applying ultraviolet light in specified patterns to form a part layer by layer by curing deposited liquid resin, which is commonly used for stereolithography printing [44,45]. In direct laser writing, ultraviolet light is directed towards a vat of photosensitive resin to form solid layers with a moving build platform (Figure 3B). Resin curing also occurs in polyjet printing, with the deposition of ink/resin on a surface with subsequent ultraviolet curing [9,17]. Powder 3D printing relies on fusing powders of a selected material using lasers in selective laser sintering [46,47] (Figure 3C) or by chemical means in binder jetting. In these processes a bed of powder is solidified and replenished layer by layer to form a part.

### 3.1. Extrusion

Among extrusion 3D printing processes, fused deposition modeling is the most commonly used (Figure 3A) [41,42]. In fused deposition modeling material is fed into the printer as a continuous filament. The extruder body is heated to melt the filament that is extruded by the pressure generated by the filament feed. After filament extrusion, the filament cools down and solidifies to form a solid geometry. Some of the most common printing materials for fused deposition modeling are polylactic acid (PLA), acrylonitrile butadiene styrene (ABS), polyethylene terephthalate (PET), and thermoplastic polyurethane (TPU). Support materials are also available that are removed during post-processing and include water-dissolvable materials such as polyvinyl alcohol (PVA), breakaway materials, and wax. The performance of the printed parts depends on material selection and process parameters such as layer thickness, build orientation, raster angle, infill density, nozzle temperature, and printing speed [48]. In fused deposition modeling, the nozzle temperature is generally maintained at a few degrees higher than the melting point of the polymer, since further increasing the nozzle temperatures may affect the performance for materials like PEEK and polyetherimide (PEI). It has been reported that the elongation percentage before failure and impact strength of a PEI part starts reducing when the temperature increases beyond an optimal nozzle temperature [49]. On the other hand, lower temperatures may result in extrusion difficulty and poor print quality due to the formation of porous volumes between the layers [49]. Additionally, layer size presents trade-offs in print resolution, part performance, and printing speed while resulting in variable amounts of anisotropy in final part properties introduced by patterning of layers in specified directions.

Direct ink writing, also known as robocasting, is another extrusion 3D printing process that avoids the heating requirements of fused deposition modeling, and rather deposits a shear, thinning viscoelastic material via a nozzle by applying external shear stress [50,51,52]. Since the process enables printing in ambient conditions, it is ideal for printing soft materials. As the shear stress increases, the viscosity of the ink reduces and enables extrusion through the nozzle. As the ink is extruded, it regains its viscosity to form a 3D structure. The filaments are stacked to additively form the final part. The printed part is cured in a different environment as per the material requirement. Direct ink writing is used to print different materials including bio-inks [43], fiber-suspended inks [50,53], electro/magnetic inks [54], and multi-material inks [55]. The capability of printing different materials in direct ink writing has made it possible to produce designs for diverse applications [50,52]. Some of the most widely used polymers for direct ink writing are polydimethylsiloxane (PDMS), thermoplastics, and epoxy. The major factors in determining the printability are the viscosity and shear thinning property of the material.

### 3.2. Resin Curing

Resin 3D printing processes expose photosensitive monomers to controlled ultraviolet light or other high energy light sources [56]. Resin curing processes typically benefit from high resolutions and quality part finishing in comparison to other printing methods in comparable price ranges. Ultraviolet curing strategies include stereolithography with direct laser writing (SLA; Figure 3B), digital light processing (DLP) [57,58], continuous liquid interface production (CLIP) [58], and continuous digital light manufacturing (CDLM) [59], which all have varied strategies of exposing a vat of resin to light to form a part. Stereolithography printing with direct laser writing includes a resin tank, a high energy light source, and a reflecting mirror to control the resin exposure to a laser. The resin in the tank is exposed to a computer-controlled laser that solidifies the resin to form a solid layer. After exposure to one layer, the printing platform moves vertically for printing the next layer [56]. After all the layers are printed, the part is washed and cured under ultraviolet light to strengthen the structure, which provides fine tuning for specific applications [60]. The duration of curing alters the printed part mechanics, for instance, when comparing parts that were post-cured for 30 h to those that had no post-curing, the post-curing with ultraviolet light was more time-efficient and improved mechanical properties, such as elastic modulus, and promoted material homogeneity through higher crosslinking [61]. Though stereolithography printing has a high resolution and printing speed, in general, it lacks multi-material printability.

Polyjet (also known as inkjet) printing is an alternate resin curing process that uses a nozzle to deposit droplets of material that are immediately cured by an ultraviolet beam upon deposition to form a layer [62]. Polyjet printing is advantageous for printing multimaterial models rapidly with multi-nozzle jetting, which also enables printing with support materials [63,64]. However, materials should generally still have shear thinning properties, which limits availability [58]. Inkjet printing has applications in fields ranging from prototyping to electronics to bio-printing [62,64], and has been demonstrated recently for use in biomedical devices using mechanically efficient lattice structures [9]. Lattices were printed using a network of beams with diameters of approximately 400µm, with fabrication defects depending on topology design and build direction. Further studies are required to determine whether polyjet printing is suitable for tissue engineering applications, with a need to further demonstrate its capabilities by producing structures with cell seeding and proliferation capabilities [30]. However, the technology provides a potential for the rapid fabrication of large sets of structures that are customizable for specific patients in applications such as safety equipment.

### 3.3. Powder Fusion

Powder fusion processes rely on depositing powder layers that are either melted or bonded to additively fabricate parts. Two common powder fusion techniques for polymer printing are selective laser sintering and binder jetting [65]. Figure 3C demonstrates the working principles of selective laser sintering, which relies on a powder stock leveled to enable fusion of one layer through exposure to a laser that follows a specified path. Once a layer is printed, the platform is lowered, and the process is repeated. One of the major advantages of selective laser sintering is the leftover powder in the platform acts as a support during part construction. Therefore the process does not require printing a separate support material and enables complex part and assembly fabrication [47].

In binder jetting printing, a jetted material binds powder as an alternative to laser melting [65]. The powder is spread on the printing platform within a predetermined thickness and then the binding material is injected to form a bonded layer. The binder jetting technique uses multiple nozzles to inject the binding material, which is potentially faster than laser melting. Binder jetting is generally an efficient process capable of printing multicolor, multi-material, and functionally graded materials [66]. Since the binding material acts as an adhesive to hold the powder together and form a printed geometry, the achieved properties of the printed parts depend on the binding material in addition to the shape and size of the powder [67,68].

## 4. Design Strategies

**Figure 4:**
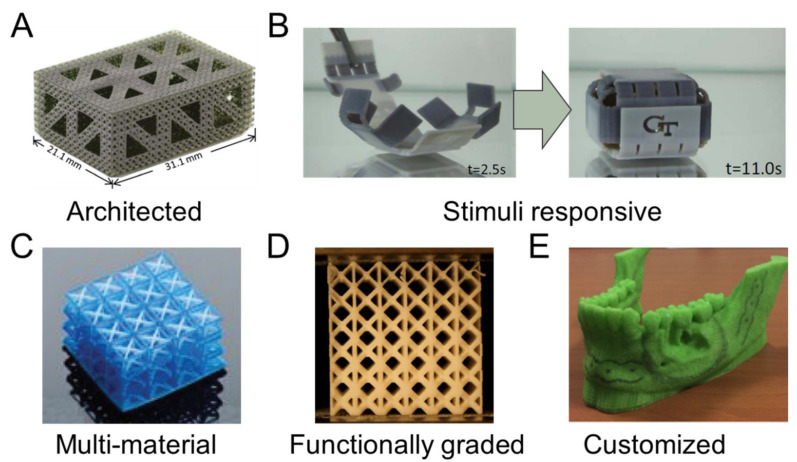
Design strategies including a **(A)** hierarchical architected lattice [14], (**B**) thermo-responsive container [15], (**C**) multi-material structure [69], (**D**) functionally graded lattice [70], and (**E**) customized mandible template [71]. Images adapted with permission.

Design strategies that are application independent provide a means for 3D-printed parts to support a desired functionality that extends beyond simply printing a solid part. Investigated strategies are presented in Figure 4 including (A) architected materials [14], (B) responsive polymers [15], (C) multi-material combinations [69], (D) functionally graded materials [70], and (E) customization [71], which all provide a means for improving the functionality and performance of printed parts. These layout strategies are beneficial for medical applications because they provide further refinement of functionality and properties for designed devices beyond the selection of materials and printing processes.

### 4.1. Architected Materials

Architected materials are designed structures engineered with a regular patterning of subunits, such as a lattice with unit cells following a designated topological distribution that takes advantage of organized material placement to improve mechanical properties for a given structural density. The synthesis of architected materials has become efficient with the emergence of 3D polymer printing that enables the printing of complex geometries with high resolution and precision [72]. The material organization throughout the structure determines the properties of the part, which are scaled from the base material properties used to construct the architected structure [73,74]. Constructing architected materials opens the possibility to engineer designs across a wide range of elastic modulus and density values through considering varied strategies for topological material distribution.

Stretch dominated beam-based lattice structures are architected materials commonly used for carrying loads in medical applications due to their high mechanical efficiency, although bending-dominated foams are also desirable for their energy absorption properties [75]. Patterning unit cells provides a simple way of configuring an architected material by first designing a single unit cell consisting of beams and then placing unit cells adjacent to one another to form a lattice structure [76]. The beam diameter and the topology of beams within a unit cell additionally inform the biological functionality of the architected material [77], such as supporting mechanobiological processes for tissue growth [5]. Hierarchical strategies are a more sophisticated approach to producing architected materials for improved mechanics [78], and are demonstrated in Figure 4A for a tissue scaffold application [14]. Hierarchical architected materials have a moderately decreased elastic moduli and a highly increased nutrient transportation capability due to larger pores introduced by the hierarchy [17], therefore providing potential performance improvements for tissue scaffolds in regenerative medicine applications.

### 4.2. Stimuli-Responsive

Stimuli-responsive designs rely on the coordinated placement of printed parts that have a directed state change when an external stimulus, such as light, heat, or force, is applied [79]. The application of external stimuli modulates the energy in the system that drives a desirable mechanical action [80]. A common strategy for stimuli-responsive parts is the combination of contrasting materials with different reaction levels to a stimulus. The combined material reactions throughout the system provide a directed response, as exemplified in Figure 2B with a combination of shape memory polymers to form a self-folding box [15]. Thermal energy was used to drive shape changes on the basis of the time-dependent behaviors of each polymer to close the box. Additionally, a single material may be cleverly distributed throughout a system to react with shape memory to form different shapes according to stimuli. Key considerations in stimuli-responsive material design are how the mechanics and interaction of materials control the change in part shape and the duration of time for responses when external stimuli are applied.

The combination of materials with contrasting levels of response to stimuli was explored recently with a glassy polymer coupled with an elastomer using extrusion printing to form a rod shaped structure [81]. Here, the glassy polymer was more prone to change shape in response to external heating stimuli. The result demonstrated that by carefully tuning the stimuli the thermomechanical behavior can induce more than 300% of the failure strain. High-resolution and high-contrast microdisplays have also been used for high-resolution photocuring that has enabled the manufacturing of 3D-architected photo-shape memory alloys [82]. In one of the studies, a new 4DMesh method was introduced using a thermoplastic actuator for shrinking and bending a 4D print to form a non-developable surface [83]. The study also validated the aesthetic, mechanical, and geometric properties of the print and demonstrated its application in industrial packaging and molds.

### 4.3. Multi-Material

Multi-material 3D prints are increasingly investigated for improving overall functionality and performance in printed parts through combining materials with contrasting properties [64]. Multi-material printing has been commonly incorporated with printing processes including fused deposition modeling, direct ink writing, and material jetting. Multi-material printing uses either a single nozzle extrusion that prints materials one at a time, or a separate nozzle for each material [50,84]. The mechanical properties of multi-material periodic composites are unique compared to single-material structures. For instance, fused deposition modeling has been used to combine a stiff periodic structure embedded in a hyperplastic material to reach a high compliancy and rate of strain recovery [85]. The performance was achieved through the embedded highly flexible matrix facilitating a uniform distribution of the applied load throughout the periodic structure, therefore enhancing the overall mechanical response. Multi-material printing has also been used to fabricate medical phantoms that reproduce mechanical properties of biological tissues while recreating anatomically accurate models [86].

The possibility of multi-material 3D printing for a functional and shape-morphing structure using direct ink writing has been recently demonstrated [87]. Multi-material printing is also advancing the field of metamaterial printing through the use of a tunable negative Poisson ratio for a uniform cell structure (Figure 4C) [69]. Instead of using the geometric parameters to control the Poisson ratio, the application of different elastic behaviors of the printed material was demonstrated by printing the beams with flexible and rigid polymers [69]. The multi-material technique has also been applied in the field of fiber-reinforced composites printing, where fiber orientation in a polymer-fiber composites was studied using direct ink writing [50]. In this study, an epoxy-resin-based ink was proposed to control the fiber orientation and demonstrated an up to 10-fold improvement in mechanical strength. Multi-material printing with multiple nozzles is an efficient and fast way of printing multiple materials simultaneously. A direct ink writing multi-material and multi-nozzle print head is able to print up to eight different types of material, with capabilities of controlling deposition of each material at the scale of individual voxels that enables printing parts for diverse applications [84].

### 4.4. Functionally Graded

Functionally graded materials are architected materials that have been engineered with a gradual geometric or material transition throughout the structure [88]. Functionally graded materials prevent the drastic transition of mechanical properties at interfaces and provide a smooth transition of properties. Thus, functionally graded materials mitigate stress concentrations over interfaces and provide durability, especially as load-bearing supports [89]. Functional gradients are additionally useful in medical applications since they provide a complex structural diversity of bioinspired gradients and facilitate more control over fluid flow, mass transport, biodegradation, and mechanical properties, such as stiffness, strength, and hardness, throughout a designed structure, which are beneficial for biomedical implants [90].

Figure 4D shows a functionally graded lattice structure where beams have varied thicknesses based on their location [70]. The structure is lightweight and has excellent energy absorbing capabilities due to its deformation behavior. The structure demonstrates deformation at the lowest density layer first, and then the deformation continues with a layer-by-layer collapse in sequence, except for the last layers that collapse concurrently or very shortly after one another. This sequential deformation of layers is enabled by the density gradient and provides desirable behaviors in mechanical responses for applications where sudden mechanical failures are a concern.

### 4.5. Customization

Customization enables printing parts with geometries altered on a per-print basis that is particularly useful for patient-specific fabrications for personalized medicine, where the configured layout matches a specific patient’s anatomy. For instance, in bone tissue engineering, implant devices are printable based on the patient’s bone geometry that has been imaged and provides a better interface to improve host-bone compatibility [91]. Such customization is additionally important for dental implants to ensure proper fits [92]. Customized layouts are also used for model printing that is representative of a patient’s unique physiology, as demonstrated in Figure 4E for a 3D-printed mandible model made of polylactic acid [71]. The printed model can aid in complex mandibular reconstruction by providing the opportunity of planning medical operations using the physical part. Planning using the 3D-printed model can help in improving the quality and precision of surgery, while also providing overall time savings.

Manual customization is often cumbersome due to the number of layout possibilities to consider when fitting a part for a patient, which is why image-based techniques are commonly applied for automated design customization [93]. For instance, a set of 2D images of a patient’s CT scan are converted to a 3D image. Then, 3D imaging data is converted to virtual 3D surface shape that is matched with optical scan data to form a 3D-printed object blueprint or a variety of other imaging techniques [94]. Further strategies by engineers can use imaging data combined with optimization techniques to create 3D-printed parts that are fine-tuned for a patient’s specific geometry, while also improving performance in comparison to traditional manufacturing processes.

## 5. Medical Applications

**Figure 5:**
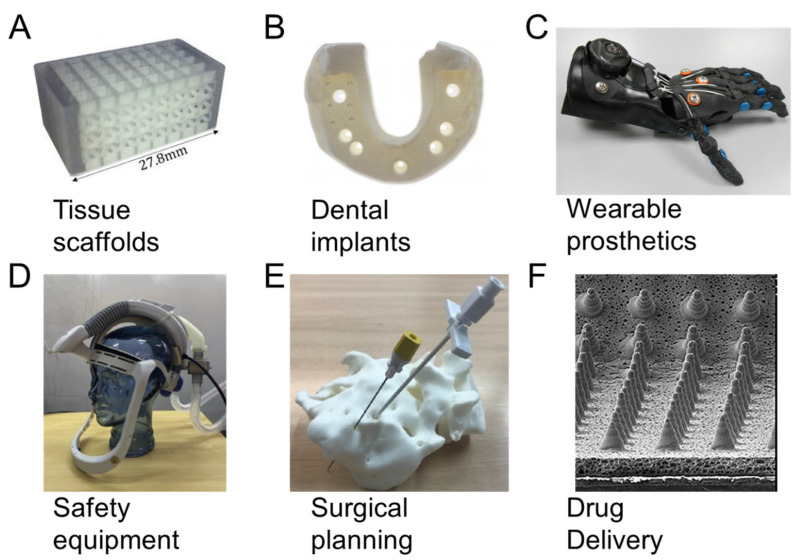
Medical 3D printing applications for (**A**) spinal fusion cage [95], (**B**) dental model [96], (**C**) prosthetic hand [97], (**D**) personal protection equipment [12], (**E**) sacral surgery planning [8], and (**F**) drug-delivering microneedles [98]. Images adapted with permission.

The consideration of materials, processes, and design strategies enables tailored 3D-printed part fabrication, which is particularly beneficial for the medical industry. Throughout Section 5, we consider how recent advances in polymer 3D printing are enabling new capabilities in medicine as demonstrated in Figure 5 for a (A) spinal fusion cage [95], (B) dental model [96], (C) prosthetic hand [97], (D) personal protection equipment [12], (E) sacral surgery planning [8], and (F) drug-delivering microneedles [98].

### 5.1. Tissue Scaffolds

3D polymer printing has recently gained interest in tissue engineering applications, where materials, process, and design strategies all play a role in the tailoring of scaffold structures [1]. Polymeric scaffolds are used in tissue engineering for synthesis of organs and have a primary purpose of restoring function or regenerating tissues [99,100]. Targeted tissues include bone, cartilage, ligament, skin, vasculature, neurons, and skeletal muscle. 3D printing is beneficial to provide personalization to patients and produce structures that are fine-tuned for clinical applications through efficient modular designs [101].

Scaffold optimization and design tuning is challenging, and in the case of bone tissue engineering, also requires the tuning of both biological and mechanical characteristics [102]. There is also the need to consider scaffold features across scales, such as hierarchical networks of pores for tissue growth and nutrient transport, with topology optimization as a commonly used configuration approach [10]. Figure 5A demonstrates a 3D-printed scaffold created with polyjet printing configured from the investigation of multiple topology layouts, beam diameter sizes, unit cell sizes, and localized reinforcements for spinal fusion applications [95]. The study used a computational approach to compare relative trade-offs among designs to find viable scaffold configurations for bone growth. Further works have investigated trade-offs using tissue growth simulations and considering asymmetric unit cell structures generated with computational design [5,76]. Computational design and automated approaches are generally useful for 3D printing applications in medicine, since designs often benefit from unique configurations for specific patients.

### 5.2. Dental Implants

There are about 276 million persons throughout the world that suffer from tooth loss and could benefit from new solutions for dental implantation [103]. The emergence of 3D-printed polymers has provided economic and precise dental implants. In these treatments, 3D-printed polymers, such as polylactic acid, are fabricated and implanted in an oral cavity since they are resistant against impact and are non-toxic [104]. 3D-printed polymers also have little surface roughness, which is beneficial since surface roughness promotes biofilm formation that attracts harmful bacteria to the implant [105]. Figure 5B demonstrates a polymer dental cast using polyjet printing from a study that compared 3D-printed dental casts to those made of dental stone; the 3D-printed cases were investigated with multiple printing processes and materials [96]. Results demonstrated that polyjet and stereolithography printing processes provided accuracies similar to conventional dental stone implants, with differences of means in measurements on x, y, and z axes being generally less than 15 µm for the best prints.

3D-printed polymers are implemented as crowns and bridges for provisional and fixed dental restoration. Fabricated crowns and bridges provide a low amount of internal discrepancies while also providing accurate occlusal fits [106]. Previously, metal structures were used as removable denture components and frameworks, but recently, PEEK polymers have replaced metals because of their high mechanical resistance with good biocompatibility [107]. Recently, researchers and medical professionals have developed and successfully implanted a patient-specific 3D-printed biopolymeric tooth [108]. The tooth was customized to the patient and provided further advantages of being high quality and low cost.

### 5.3. Wearable Prosthetics

3D printing offers a wide variety of approaches for new prosthetics that benefit from a range of material availability and customization for a person’s needs. In Figure 5C, a 3D-printed prosthetic hand is demonstrated that is a combination of PLA and ABS materials for children with upper limb issues [97]. The wearable hand is low cost and provides a broad range of motion for users. Stretchable prosthetics with embedded actuators, signal processors, and sensors have also been tailored for individuals [109,110]. For instance, a smart wearable therapeutic device was fabricated with an embedded temperature sensor and programmable heater for self-activation according to a patient’s body temperature [111]. Recently, a pressure sensor-integrated 3D-printed elastomer-based wearable device was developed [112]. The device detects and monitors human body movement, external pressure, and the direction of external forces, which implies its potential as an electronic skin.

Every year, hundreds of thousands of people suffer from spinal cord injury around the world who could benefit from prosthetics [113]. Spinal cord injury can affect hand-function and locomotion. A polylactic acid (PLA) based 3D-printed wearable hand orthosis has been designed and fabricated to aid patients [114]. The device acts on the electromyography signal and works for the grasping function of the patient. Bone fracture is another prevalent medical problem where high density polyethylene (HDPE) or polypropylene (PP) based 3D-printed personalized wearable casts have been proposed and implemented for successful bone recovery [115].

### 5.4. Safety Equipment

The 2020 COVID-19 pandemic has elevated the importance of polymer 3D-printed safety equipment, as the conventional safety equipment supply was inadequate in certain regions when the need for personal protection equipment vastly exceeded demand. Polypropylene 3D-printed particle filters and masks were proposed as an alternative resource to help meet demand and avoid supply chain issues [116]. Additionally, in one study, a 3D-printed respirator was developed using TPU, ABS, and PLA filaments [13]. This respirator was reusable, easy to clean, and usable with an arbitrary number of filtration units. Figure 5D demonstrates a 3D-printed helmet for use as personal protection equipment [12]. The primary helmet component integrates a breathing filter with a conventional safety helmet to provide an efficient means of creating safety equipment locally.

Studies have confirmed that 3D-printed architected materials are usable as helmet liners for protection from head injuries and provide advantageous energy absorption performance [117]. The energy absorption capabilities are tunable by using functionally graded materials. Architected helmet liners perform well for the multi-impact loading that is commonly experienced during motorcycle crashes [118]. Helmet testing has demonstrated that the liners have achieved standards for impact testing, while design variations in hole sizes provide tuning for optimal performance.

### 5.5. Surgical Planning

Surgical planning models have been 3D printed with rigid plastics including PLA and ABS for visualizing patient-specific organ models prior to operation. 3D-printed organ models are fabricated on a patient-specific basis at low cost, and have been applied in several medical fields including cardiology [119,120], neurology [121,122], urology [123,124], and osteology [8,125]. Figure 5E demonstrates a patient-specific 3D-printed sacral model using PLA [8]. This model is used for refining techniques for sacral anomalies and for training new surgeons.

ABS filaments have been used in cardiology to fabricate the anatomical structure of patient-specific hearts for improving inflow in a device implantation procedure [120,126]. Studies have also fabricated anatomically accurate 3D-printed models for the pulmonary trunk and ventricular outflow tract using thermoplastic polyester resins [127]. PLA filaments and photosensitive liquid resins have been used to fabricate 3D-printed aneurysm models with hollow craniums and rigid walls [121,122]. These aneurysm models replicate patient-specific anatomies used to study the hydrodynamics in the system. Rigid photopolymers have been implemented to fabricate 3D-printed kidney models and prostates [123,128]. Patient-specific modeling was also conducted for a kidney with a removable tumor [129]. As a whole, these printing applications provide surgeons a way to experience and plan a surgery in a minimally invasive way prior to performing an actual surgery.

### 5.6. Drug Delivery

3D-printed drug delivery enables the fabrication of drugs for patient-specific needs, uniform drug distribution, and solvent-free drug-containing material production [130]. 3D-printed polycaprolactone and tricalcium phosphate meshes have demonstrated that micro-architecture influences drug delivery efficacy [131,132]. In vivo and in vitro studies demonstrate that these drug delivery constructs are resistant against Gram-positive and Gram-negative bacteria, while also potentially delivering a higher percentage of the incorporated drug to the body.

Drug delivery is also possible through application of 3D prints outside of the body. Figure 5F demonstrates a 3D-printed microneedle array that drives drugs directly through the skin for microcirculation in the body [98]. These delivery approaches generally remain pain-free while promoting efficient transport that requires sophisticated geometric fabrication at a microlevel enabled by 3D printing. The microneedles are fabricated with a tip width between 65 and 84 µm, a pitch of 700 µm, and heights between 422 and 481 µm.

Polymeric 3D printing is also applied for fabricating drug delivery systems with multi-active dosage forms [133], time-tailored release tablets [134], and multilayer caplets [135]. The technology has been demonstrated for personalized drug delivery that can control release rate, drug combination, and dosing intervals [136]. Dosing requirements vary in patients based on their physiological functioning, which motivates personalization to improve patient responses. 3D-printed polymeric microcapsules and nanocapsules remain stable in the liquid suspension and biological fluids that improve drug efficiency [137], thereby motivating their use for controlled drug release.

## 6. Outlook

**Figure 6:**
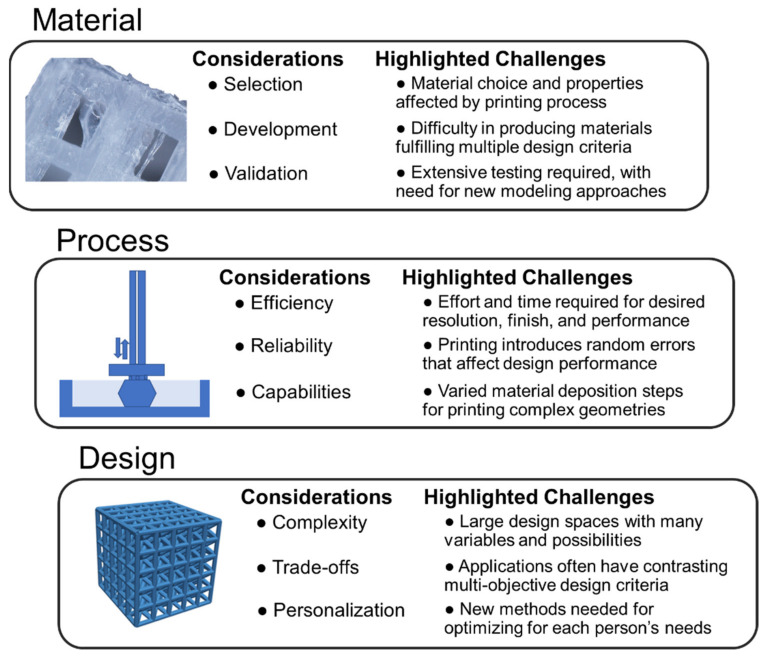
Key research challenges for 3D printing polymers using materials, process, and design strategies for medical applications.

Although there are numerous successes in translating polymer 3D printing research to medical applications, many challenges remain. Some of the key considerations in advancing research in material, process, and design strategies for 3D polymer printing are presented in Figure 6, which additionally includes highlighted areas for researchers to address. Although issues are separated by material, process, and design, there is a large overlap between factors, such as material properties being influenced by process parameters and design performance being dependent on fabrication consistency. The development of new, integrated design strategies and computational approaches that holistically consider materials, process, and design in the development of new products is therefore essential for improving 3D-printed polymer performance in medical applications.

Though numerous 3D printable polymer materials have been developed in recent years, it is not always straightforward to select materials for specified applications. Material selection is a critical aspect of achieving a print with desirable properties, but it is challenging, since 3D-printed polymers have uncertainty and ranges in performance based on the printing process and parameters [138] For example, parts printed with fused deposition modeling exhibit anisotropy with higher mechanical strength in the printing direction compared to the transverse direction [49]. Additionally, applications often require materials with multiple properties, such as the need for highly stiff materials for bone tissue engineering that also exhibit biocompatibility for tissue engineering [1]. The need for both material capabilities simultaneously limits the scope of possible materials. For instance, the polymer selected must have suitability for in vivo implantation, which creates difficulties when considering photopolymer resins that are potentially toxic if not fully cured, and many other materials with desirable mechanics that are not biocompatible.

Design strategies can help mitigate these material deficiencies, possibly through combining two contrasting biocompatible materials or through clever architected configurations to reach an overall beneficial, global system performance. When combining materials or considering the development of new materials, there are rigorous requirements of testing and validation of the printed parts, especially when considering the need for testing for all print process parameter combinations [139]. Improved modeling approaches could aid in predicting part performance to reduce the number of experiments required, which could benefit from new computational approaches and efficient design of experiments.

Research and advancement of suitable printing processes is also necessary due to the limitations and trade-offs associated with each 3D printing approach. For instance, there is an inherent trade-off for all printing processes between resolution, printing speed, and fidelity that affects the manufacturing logistics, mechanical performance, and surface finish of printed parts [140,141]. Generally, as resolution is increased, the quality of a part improves but requires more time to print. The trade-off between resolution and printing time differs on the basis of the printing process. For instance, processes such as fused deposition modeling and direct laser writing stereolithography, which have to follow a specified path to solidify each layer of a part, have print duration scaling according to part size and number of parts, whereas stereolithography processes, which project light that solidifies an entire layer of material at once in a resin vat, have print duration scaling with only the height of the part. Post-processing such as washing, curing, and support removal can also affect production times and performance [61]. In general, fused deposition modeling and selective laser sintering do not require washing and curing, while stereolithography requires washing to remove liquid resin and post-curing that additionally affects part mechanics. Support material removal can also increase part finishing time, resulting in broken parts or reduced performance from introduced cracks. Selective laser sintering and other powder processes may require brushing to remove excess powder from parts, which increases post-processing time and requires extra equipment such as powder removal stations. Further advancements in multi-nozzle printers could potentially improve production speeds while retaining part quality, or as the technology continues maturing, prices could drop that enable greater amounts of simultaneous part printing, since some printing speeds are limited by physics dictating how fast materials can be deposited and solidified, which often cannot be increased to improve speeds for single part production.

Printing reliability is crucial for ensuring parts operate as expected and to reduce the need for disposal of parts printed with major defects. Printing processes can introduce defects related to residual stress [142], porosity [143], and impurities [144]. The residual stress can cause permanent deformation, warping, delamination of layers, and lack of adhesion to the build plate. These process limitations indicate that research studies should be done on reducing fabrication errors that can lead to the near-error-free fabrication of 3D-printed polymer parts suitable for sensitive microscale medical devices. Further, for ABS fused deposition modeling parts, build orientation has been additionally demonstrated to influence reliability, which means design decisions for part layouts will also affect print process outcomes [145]. Fabrication errors can emerge from layouts of filament to form solid structure and are subject to greater possibility of failure around areas that lead to stress concentrations or geometries, such as holes that lead to the poor bonding of filaments.

The selection of a printing process for an application is also driven by their capabilities for printing specified geometries [138]. In general, extrusion processes have difficulties in forming overhangs that are necessary for many lattice structures, resin processes require support material or self-supporting geometries [146], while powder processes have unused powder that supports parts during printing to promote complex geometry formation [47]. Some support material strategies in extrusion printing and polyjet printing enable more complex part geometries than otherwise possible, but require further post-processing and the possibility of damaging prints during support removal [147]. Fused deposition modeling tends to be a cheaper process that can produce parts with sound mechanical performance [148]. Direct ink writing is suitable for printing in ambient conditions [50], although it has limitations in tuning part performance during printing since there is no heating involved. Resin printing processes are known for having high resolution, surface finish, and printing speed [138,149]. However, stereolithography printing lacks multi-material functionality, while polyjet printing leads to inconsistent surfaces for parts printed at its resolution limits [30]. Powder printing techniques are suitable for printing entire assemblies with powder acting as support, while being generally more expensive and having resolution limits based on the powder particle size. These considerations suggest a need for suitable design strategies for applications that maximize performance for prints based on the strengths and limitations of each printing process, and a need for improved printing processes and methods to bypass the inherent deficiencies of each process.

The design strategy selected for an application requires consideration of available materials and processes in relation to the medical application of interest. If complex geometries are necessary for constructing lattices or anatomically complex models, then stereolithography or powder processes may work best. If multiple materials are necessary, then fused deposition modeling or polyjet printing may be the most appropriate. Although it is possible to print complex geometries with multiple processes, it is difficult to determine an optimized configuration because of the wide-ranging possibilities in material choice, process parameters, and design decisions. For instance, a multi-material lattice structure may consist of thousands of beams that could all have individually specified materials and diameters [150]. New computational and experimental methods are necessary to aid design approaches for finding optimal solutions and fully leveraging 3D printing technologies [9]. Some researchers have developed Voronoi lattices to introduce bio-mimetic morphological scaffolds that can be fabricated by polymer 3D printing, which necessitates new tools for tuning Voronoi lattices for specific trade-offs [151,152]. Further considerations that could improve design opportunities are combinations of varied strategies, such as architected multi-material strategies or the creation of 3D-printed assemblies from different processes to leverage the strengths and limitations of each approach.

Engineering design approaches are necessary for navigating the multi-objective trade-offs common in medical engineering applications, such as mechanical and biological functionality in regenerative medicine [1,5]. Solving multi-objective problems requires weighing the importance of different variables for tuning a design for higher or lower performance in different situations. Such trade-offs are also inherent to navigate in 3D printing processes between speed, time, and mechanical performance, which are affected by fabrication artifacts. Finding high-value solutions in complex design space searches remains a challenge for 3D printing applications that could benefit from computational design approaches with improved navigation of search spaces [153]. Computational approaches could also aid in predication of part performance which is necessary for design search evaluations. For instance, the construction of multi-material parts has opened new possibilities in combining materials to create entirely new systems that operate differently than their individual components, and may exhibit advantageous emergent behaviors. Design customization on a per-print basis opens new doors for personalized medicine, but again, there is a need for new computational approaches for automatically tuning designs to a patient’s specified needs [154]. Future advances in design are necessary to aid nonlinear and integrated decision making across materials and processes for specified applications, where there is much room to explore new methods for fully leveraging the capabilities 3D polymer printing.

## 7. Conclusions

A review of polymer 3D printing for medical applications was conducted, and highlighted how material, process, and design decisions influence application performance, therefore necessitating designers to carefully consider all factors when configuring parts. Recent research has demonstrated a diversity of polymer materials with varied properties according to 3D printing processing parameters. Design strategies enable the directed placement of materials to achieve improved performance with configurations such as architected or multi-material structures. Because of the complexities involved in considering all factors that influence application performance, it is recommended that researchers conduct further experiments considering the interactions of materials, processes, and design strategies, while developing new methodologies to handle decision making and configuration for applications. Overall, advances in 3D polymer printing have demonstrated many successes for implemented designs, with a need for continued research to fully leverage the technology for wide-ranging applications in engineering and medicine.

## Figures and Tables

**Table 1:** Measured 3D-printed part properties organized by material and printing process. Further details included to provide relevant context.

Material	Printing Process	Measured Properties	References
Acrylonitrile butadiene styrene (ABS)	Fused deposition modeling	Tensile Strength: 35 MPa; Elastic Modulus: 1300 MPa.	[36]
Acrylonitrile butadiene styrene (ABS)	Fused deposition modeling	Tensile Strength: 27–31 MPa; Layer height: 0.05–0.14 mm; Processed at 210–240 °C.	[34]
Acrylonitrile butadiene styrene (ABS)	Fused deposition modeling	Tensile Strength: 15–38 MPa; Elastic Modulus: 1220–1430 MPa; Orientations of 0° to 90°.	[35]
Polycarbonate (PC)	Fused deposition modeling	Tensile Strength: 37 MPa; Elastic Modulus: 1000 MPa.	[36]
Polycarbonate (PC); Biomaterial blend	Fused deposition modeling	Tensile Strength: 35–65 MPa; Elastic Modulus: 2100 MPa; Nozzle Temperature: 240–270 °C; Orientations of 0° to 90°.	[35]
Polycarbonate (PC); Fossil-fuel blend	Fused deposition modeling	Tensile Strength: 28–62 MPa; Elastic Modulus: 1300–1500 MPa; Orientations of 0° to 90°.	[35]
Polyether ether ketone (PEEK)	Fused deposition modeling	Tensile Strength: 58–85 MPa Elastic Modulus: 3000–4100 MPa; Temperature dependent.	[37]
Polyethylene terephthalate glycol (PETG)	Fused deposition modeling	Tensile Strength: 36–40 MPa; Layer Height: 0.05–0.14 mm; Processed at 210–240 °C.	[34]
Polylactic acid (PLA)	Fused deposition modeling	Ultimate Strength: 265 MPa; Yield Strength: 205 MPa; Elastic Modulus: 4400 MPa; Compression Testing.	[38]
Polylactic acid (PLA)	Fused deposition modeling	Tensile Strength: 28–56 MPa; Elastic Modulus: 2000 MPa; Orientations of 0° to 90°.	[35]
Polyamide 12 (Nylon)	Multi jet fusion	Tensile Strength: 47–48 MPa; Elastic Modulus: 1150–1250 MPa; Orientations of 0° to 90°.	[39]
Acrylic-based (Stratasys: MED 610)	Polyjet	Elastic Modulus: 1860–2120 MPa; Compression Testing; Orientations of 0° to 90°.	[9]
Epoxy-based (DSM Somos, Inc: Watershed XC 11122)	Stereolithography	Tensile Strength: 37–48 MPa; Elastic Modulus: 2040–2400 MPa; Orientations of 0° to 90°.	[40]
Methacrylic Acid (EnvisionTEC: E-Shell 600)	Stereolithography	Elastic Modulus: 1400–1620 MPa; Compression Testing; Orientations of 0° to 90°.	[16]
Methacrylic Acid (Formlabs: Dental SG)	Stereolithography	Elastic Modulus: 1670 MPa; Compression Testing.	[17]
